# The association of parental involvement with adolescents’ well-being in Oman: evidence from the 2015 Global School Health Survey

**DOI:** 10.1186/s40359-021-00677-5

**Published:** 2021-11-08

**Authors:** Tehniyat Baig, Gowrii S. Ganesan, Hania Ibrahim, Wajiha Yousuf, Ziyad R. Mahfoud

**Affiliations:** 1 Department of Medical Education, Weill Cornell Medicine - Qatar, Doha, Qatar; 2 Division of Epidemiology, Department of Population Health Sciences, Weill Cornell Medicine, New York, NY USA

**Keywords:** Adolescent health, Parental involvement, Oman, GSHS

## Abstract

**Background:**

The parent-adolescent relationship plays a key role in adolescent development, including behaviour, physical health, and mental health outcomes. Studies on the parental factors that contribute to an adolescent’s dietary habits, exercise, mental health, physical harm and substance use are limited in the Middle East and North Africa region, with none in Oman. This study aims to investigate the association between parental involvement and adolescent well-being in Oman.

**Methods:**

Cross-sectional data from the 2015 Global School Health Survey for Oman was analysed. The dataset consisted of 3468 adolescents. Adolescents reported on their parental involvement (checking to see if they did their homework, understanding their problems, knowing what they are doing in their free time and not going through their things without permission). Parental involvement was scored on a 20-point scale. Associations with the following dependent variables: nutrition, exercise, hygiene, physical harm, bullying, substance use, tobacco use and mental health well-being were done using Spearman’s correlations, linear and logistic regressions.

**Results:**

The surveyed population was 48% male, 65% aged 15 to 17 years old and 5% reported that they “most of the time or always” went hungry. Parental involvement was positively correlated with each of the dependent variables. Adolescents with higher parental involvement had significantly higher odds of good nutrition (1.391), hygiene (1.823) and exercise (1.531) and lower odds of physical harm (0.648), being bullied (0.628), poor mental health (0.415), tobacco use (0.496) and substance use (0.229).

**Conclusions:**

Parental involvement plays a positive role in all aspects of adolescents’ well-being in Oman. Awareness campaigns and interventions aimed to help improve the well-being of adolescents should incorporate such positive role in their designs.

## Background

Adolescence is the stage of life where a child transitions into adulthood and undergoes simultaneous physical, mental, and social developmental changes. According to the World Health Organization (WHO), an adolescent is an individual between the ages of 10–19 years [1]. An adolescent’s psychosocial and physical development is very vulnerable and complex as it is a period of time where they are more likely to experiment with psychological, physical and social boundaries which can significantly impact development and maturation into adulthood [28]. Recent evidence indicates that a variety of parental involvement factors such as parents’ warmth, supportive parenting, parental encouragement, and overall parental involvement were associated with improved health outcomes among adolescents [11]. Parental involvement is defined as the parent’s active participation and interaction with their child and their school [42]. This includes frequency and quality of actual involvement, monitoring of adolescent activities and development of effective communication strategies [29]. The amount and type of involvement a parent has with their child can result in a positive or negative impact on their child’s physical, mental and social development—the goal being for parents to have positive influence in their children’s lives while fulfilling the adolescent need for autonomy [43]. Studies such as De Goede [12], Keijsers [22], Van Doorn [45] have explored longitudinal models to identify patterns and key factors that play a role in adolescent development [12, 13, 22, 26] which include the effect of parental influence on adolescent behaviour and maturation. Adolescent perception of parental factors was also found to contribute to improved outcomes in a number of avenues including mental health, substance abuse, and self-esteem [8]. Further exploration of the role of parental involvement on the health and wellness of adolescents can provide valuable insight to guide clinical practice and interventions [1, 28].

The relationship between parental influence and adolescent mental health has been well documented [42]. A systematic review on the relationship between parental factors and depression and anxiety disorders in adolescents found that factors such as “less warmth, more inter-parental conflict, over-involvement, and aversiveness” were associated with an increased risk for depression and anxiety [49]. Given the especially high prevalence of depression and anxiety among this age group, exploring this potentially modifiable association may prove highly beneficial in parental counselling and clinical practice among adolescents suffering from mood disorders [42]. A recent study conducted in Sub-Saharan Africa found that low rates of parental involvement were associated with poor adolescent mental health outcomes [6]. Negative parental involvement may work opposingly where in some cases help and guide adolescents in coping with different stressors, and in some cases could be a cause of such stressors [37].

The parent–child relationship remains an undeniable influence over the child’s development into adulthood, although this time period is also when adolescents are significantly influenced by their peers [18]. One negative social interaction that may modify adolescent development is bullying. Bullying is a threatening and aggressive behaviour leading to the victimization of many school-going children and adolescents [2]. Studies have shown that adolescent bullying has a great impact on adolescent development as it can lead to future difficulties such as depression, aggression, hyperactivity/inattention, and even impaired cognitive abilities [9]. Interestingly, a systematic review on protective factors that interrupt the continuity from school bullying to later internalizing problems, identified support from family and friends as protective factors towards emotional resilience against victimisation [44]. Studies also showed that decreased emotional resilience led to higher risk of substance use, and that less parental involvement increased overall risks of risky behaviour in later adolescence [14, 35]. Furthermore, t having “warm parents, family support, and parental attachment” were significant resilience factors found to be protective against school bullying with adolescents reporting lower levels of loneliness, suicidal ideation and depression especially compared to peer to peer relationships [1, 30, 36]. Existing studies on adolescent high risk behaviour such as substance use, interpersonal violence demonstrated their association with decreased parental involvement and poor mental health, especially among middle and low income countries, however, further studies are needed to explore other contributors to mental health such as nutrition and physical activity [17, 37]. Further exploration of this association may aid in developing strategies for safer classroom environments through bullying prevention, and lead to reduction of adolescents engaging in risky behaviour in the future.

Parental involvement has also been found to be associated with adolescent physical health. Childhood and adolescent obesity are rapidly becoming a global epidemic [20]. Multiple studies have investigated the effect of parental factors on adolescent weight and physical activity, mostly showing poor parental influence leads to poorer nutritional habits and poorer exercise habits that persist into adulthood [50]. A systematic review study on the relationship between parental and child obesity found that there was a parent–child association in obesity, with stronger associations noted in older children [47]. However, a meta-analysis on the effectiveness of parent-focused obesity interventions found no significant difference in BMI with parental involvement versus without it [15]. Poor hygiene was found to be associated with other poor health habits and with poorer quality of life measures [16]. Another study found that parental support and supervision was protective for hand hygiene practices [21].

Oman is a culturally diverse country with many potential behaviors being influenced by parental involvement, this study aims to investigate the potential relationship between parental involvement and adolescent physical and mental health in Oman. One study in Oman showed that over a period of 10 years, there were increases in the prevalence of high-risk health behaviours, including increase in poor nutrition, physical inactivity, violence and hygiene. This indicates that further exploration of potential protective interventions is needed [39]. While the contribution of parental involvement to social, mental, and physical health may seem intuitive, variation in existing literature suggests that this important variable requires further investigation [23, 48, 51]. Studies looking at the association between adolescent-parent relationships and adolescents’ well-being are limited in the Middle East and North African (MENA) region and mainly focus on individual behaviours. To the best of our knowledge, there are no similar studies looking at the effects of parental involvement with adolescent well-being in Oman, with a general scarcity of literature specific to Omani adolescents. Although much literature exists using the Global School Health Survey, our study is important since country-specific differences might affect association outcomes, whereas existing studies on similar subjects tend to group countries together and as such might not be as generalizable to the general population. We hypothesize that there is a positive association between parental involvement and outcomes of adolescent health, with higher levels of involvement linked to improved health outcomes and better behaviour due to a reciprocal positive response [34, 35, 37]. As such, defining and quantifying parental involvement to determine which forms of involvement are helpful to adolescents will provide clarity amidst varying results of the current literature which could be relevant in initiating and promoting parental programs and improved integration of parental involvement within the clinical setting in Oman.

## Methods

### Study sample and data collection

Oman is one of the six Middle Eastern countries in the Gulf Cooperation Council (GCC). While the region is known for economic prosperity and a high GDP per capita, Oman is marked by its unique financial and cultural background among its counterparts. This small desert nation has a unique cultural heritage combined with a large immigrant population [37]. Arabic is the official language and the predominant religion is Islam (85.9%). Children between the age of 0 to 14 make up the largest portion of the Omani population at 30.1%. The country has 4.8 million inhabitants, 45% of which are immigrants. While Oman is a high-income country, its economic background makes it markedly different from neighboring gulf countries. Oman’s Gross Domestic Product (GDP) per capita is $46,000, as compared to $124,100, $68,600, $54,500 in neighbouring Qatar, UAE, and Saudi Arabia respectively [27]. Primary school enrollment is reported to be 100% in Oman, with a literacy rate of 96.1% at 15 years of age [31].

This is a secondary data analysis using data from the Global School Health Survey (GSHS) for Oman, collected in 2015 [32]. The GSHS is a school-based survey administered to students enrolled in secondary school, with most students between 13 and 17 years of age, used primarily for obtaining self-reported data on health behaviors that affect major causes of morbidity and mortality among adolescents worldwide. The survey used two-stage cluster sampling design and included any school containing 8th to 12th grade classes. Ethical approval was obtained from the Research, Ethical Review and Approve Committee at the Ministry of Health in Oman. Parents and adolescents were sent a letter briefing them about the study and explaining that participation is voluntary.

The questionnaire consisted of 59 self-administered questions covering a number of topics: diet and exercise habits, drug use and exposure, hygiene, mental well-being, tobacco exposure and use, physical harm, violence and unintentional injury. From a total of 63 schools, 3468 students completed the survey and the response rate from schools and students was 98% and 94%, respectively. Responses from parents were not available from this questionnaire.

The following demographic variables were collected: age, sex and ‘how often did you go hungry in the past 30 days’. Hunger as a food security measure was used as a proxy for socioeconomic status. Of note, this was not the original intended purpose of the hunger question.

### Independent variable: parental involvement

Parental involvement score utilized the four available questions regarding this topic in the database:

1. During the past 30 days, how often did your parents or guardians check to see if your homework was done?
2. During the past 30 days, how often did your parents or guardians understand your problems and worries?
3. During the past 30 days, how often did your parents or guardians really know what you were doing with your free time?
4. During the past 30 days, how often did your parents or guardians go through your things without your approval?

Respondents answered each question using a 5-point Likert scale with the following options: Never, Rarely, Sometimes, Most of the Time, and Always. Each response was given a numerical value. For questions one to three, the coding was as follows: Never = 1, Rarely = 2, Sometimes = 3, Most of the Time = 4, and Always = 5. For question four, Never = 5, Rarely = 4 Sometimes = 3, Most of the Time = 2, and Always = 1. The parental involvement score was the sum of the answers on the 4 questions and could range from 4 to 20 with higher scores indicating increased parental involvement. The median score on the parental involvement was then used to dichotomize the scale into high versus low parental involvement.

### Dependent variables: adolescent mental well-being and physical health

A number of outcome scales were created to assess the overall mental and physical well-being of adolescents in this study. Each scale was based on survey questions related to its domain. These scales were nutrition (4 questions), exercise (4 questions), hygiene (4 questions), physical harm (3 questions), bullying (1 question), substance use (2 questions), tobacco use (2 questions), and mental health well-being (5 questions). All questions had Likert scale responses as described for the parental questions above. Coding was adjusted to allow higher scores to represent more favorable behaviour or better health. As such, higher scores on nutrition and on bullying represent better nutrition and less bullying respectively. Similar to the parental involvement scale, the median score for each outcome scale was used as a cut-off point to divide the population into high and low categories.

As an example of the individual scales used for the dependent variables, nutrition was determined using the following 4 questions:

1. During the past 30 days, how many times per day did you usually eat fruit, such as dates, apples, oranges, or bananas?
2. During the past 30 days, how many times per day did you usually eat vegetables, such as tomatoes, cucumbers, carrots, or lettuce?
3. During the past 30 days, how many times per day did you usually drink carbonated soft drinks, such as Pepsi, Coca Cola, or Mountain Dew? (Do not include diet soft drinks.)
4. During the past 7 days, on how many days did you eat food from a fast food restaurant, such as burger places, pizza places, or shawarma places?

Questions 3–4 were reverse coded so that higher consumption of carbonated drinks and fast food would result in a lower overall nutrition score. Scores ran from 0 to 29 based on the frequency of consumption of various food groups, with 0 representing the least healthy diet and 29 representing the most healthy diet.

### Statistical analysis

Participants’ demographics and their self-reported answers to the parents involvement questions were summarized using frequency distributions. The association between parental involvement and adolescent behavioural and well-being scales were assessed in several ways.

First, Spearman’s correlations were used to measure the potential association between the scores of the several outcomes and the that of the parental involvement. Univariate and multiple linear regression were used to assess the relationship between each of the outcomes and the parental involvement scale as an independent predictor while adjusting for age, sex and food insecurity. Unadjusted and adjusted slopes along with *p*-values were reported. Finally, bivariate and multiple logistic regressions were fit for each of the dichotomized outcomes to assess the potential association with parental involvement (high/low) and adjusting for age, sex, and food insecurity. Unadjusted and adjusted odds ratios along with their 95% confidence intervals were presented. All data analyses were done using IBM SPSS (version 26, Armonk NY). A *p*-value of 0.05 or less was considered significant.

## Results

## Demographic characteristics of participants

The final analysis included 3486 students as shown in Table 1. The majority of the participants were between 15 and 17 years old (65%) and females made up 52% of the sample. About 5% of the participants reported that they went hungry (proxy SES) “most of the time” or “always”.

**Table 1:** Demographic characteristic of the participants

Variable	N	%
*Age*		
11 years old or younger	40	1.20
12 years old	39	1.10
13 years old	358	10.40
14 years old	531	15.40
15 years old	741	21.40
16 years old	760	22.00
17 years old	731	21.20
18 years old or older	256	7.40
*Sex*		
Male	1623	47.70
Female	1781	52.30
*How often went hungry in the past 30 days? (Proxy for SES)*		
Never	2130	61.80
Rarely	619	18.00
Sometimes	530	15.40
Most of the time	104	3.00
Always	62	1.80

About 49%, 41% and 42% of adolescents reported that parents always or most of the time checked their homework, understood their problems and knew what they were doing during their free time respectively. About 85% reported that their parents never or rarely go through their things (Table 2).

**Table 2:** Frequency distribution of responses to the four questions that comprise the parental involvement scale

Question	Responses									
	Never		Rarely		Sometimes		Most of the time		Always	
	n	%	n	%	n	%	n	%	n	%
Parents check homework	636	18.9	482	14.3	593	17.6	501	14.9	1160	34.4
Parents understand problems	814	24.3	534	15.9	649	19.4	492	14.7	864	25.8
Parents know about free time	655	19.3	571	16.8	737	21.7	575	16.9	860	25.3
Parents go through their things	2416	70.9	480	14.1	271	8.0	133	3.9	107	3.1

Descriptive statistics for parental involvement scale and other scales are presented in Table 3 with the median indicating the cut-off scores for high-low dichotomization. Scores on the parental involvement scale ranged between 4 and 20 with a median of 14. High parental involvement (n = 1814) was defined as a score greater than 14 on the tabulated parental involvement scale and low parental involvement (n = 1432) was a score equal to or less than 14.

**Table 3:** Descriptive statistics for the parental involvement and adolescent behaviour, and well-being scales

Scale	Median score	Mean score	Standard deviation	Minimum	Maximum
Parental involvement	14.00	13.96	3.62	4.00	20.00
Nutrition	18.00	18.18	3.74	4.00	29.00
Hygiene	17.00	16.70	2.82	4.00	21.00
Exercise	13.00	13.33	4.23	4.00	28.00
Physical harm	23.00	21.51	3.34	3.00	24.00
Bullying	7.00	6.18	1.31	1.00	7.00
Mental health	13.00	16.27	2.99	5.00	21.00
Tobacco use	14.00	13.68	1.36	2.00	14.00
Drug use	10.00	9.90	0.65	2.00	10.00

Spearman’s correlation demonstrated that parental involvement was significantly positively correlated with nutrition (r = 0.112, *p* < 0.001), hygiene (r = 0.208, *p* < 0.001), exercise (r = 0.133, *p* < 0.001), physical harm (0.130, *p* < 0.001), bullying (0.125, *p* < 0.001), mental health (0.200, *p* < 0.001), tobacco use (0.053, *p* < 0.001) and drug use (0.048, *p* < 0.001) as shown in Table 4.

**Table 4:** Unadjusted and adjusted slopes for parental involvement as independent variable for the different behaviour and wellbeing outcome scales

Outcome scales	Nutrition	Hygiene	Exercise	Physical Harm	Bullying	Mental Health	Tobacco Use	Drug
*Use*								
Slope for parental involvement								
Unadjusted	0.112	0.244	0.119	0.159	0.155	0.210	0.093	0.079
Adjusted++	0.112	0.208	0.133	0.130	0.125	0.200	0.053	0.048
*p*-value	< 0.001*	< 0.001*	< 0.001*	< 0.001*	< 0.001*	< 0.001*	< 0.001*	< 0.001*
N	3140	3080	3120	2893	3126	3033	3089	3031

++Adjusted for age, sex, and how often went hungry (proxy SES)

^*^Significant at the 5% level

In multivariate linear regression, adjusting for age, sex and frequency of going hungry, there were significant positive associations between the parental involvement scale and all other well-being scales. This indicates that more parental involvement is associated with better well-being of adolescents.

Table 5 shows that results from multivariate logistic regression analysis showed similar trends. Adolescents with higher parental involvement had significantly higher odds of good nutrition (aOR = 1.391, 95% CI: 1.202–1.610), hygiene (aOR = 1.823, 95% CI [1.571, 2.115]) and exercise (aOR = 1.531, 95% CI [1.321, 1.774]). They also had lower odds of physical harm (aOR = 0.648, 95% CI [0.554, 0.757]), being bullied (aOR = 0.628, 95% CI [0.541, 0.730]), poor mental health (aOR = 0.415, 95% CI 0.355–0.484), tobacco use (aOR = 0.496, 95% CI [0.360, 0.682]) and substance use (aOR = 0.229, 95% CI [0.120, 0.440]).

**Table 5:** Unadjusted and adjusted odds ratios of the outcomes in the adolescent group with “high” parental involvement as compared to “low” parental involvement

Scale	Unadjusted OR (95% CI)	Adjusted OR++ (95% CI)
Nutrition	1.407 (1.222, 1.620*)	1.391 (1.202, 1.610*)
Hygiene	1.967 (1.704, 2.271*)	1.823 (1.571, 2.115*)
Exercise	1.478 (1.283, 1.704*)	1.531 (1.321, 1.774*)
Physical harm	0.595 (1.283, 1.704*)	0.648 (0.554, 0.757*)
Bullying	0.568 (0.492, 0.656*)	0.628 (0.541, 0.730*)
Mental health	0.400 (0.345, 0.463*)	0.415 (0.355, 0.484*)
Tobacco use	0.384 (0.284, 0.518*)	0.496 (0.360, 0.682*)
Substance use	0.161 (0.085, 0.304*)	0.229 (0.120, 0.440*)

++Adjusted for age, sex, and how often went hungry

^*^Significant association at the 5% level

## Discussion

This study found limited self-reported parental involvement amongst adolescents in Oman. Nonetheless, parental involvement remains significantly associated with multiple markers of adolescent health including less risky behavior and better mental and physical well-being. Our findings suggest that increased parental involvement is significantly associated with improved nutrition, hygiene and exercise in adolescents. Increased parental involvement is also associated with lower risk of physical harm, bullying, tobacco and substance use, and poor mental health amongst adolescents in Oman. Findings of this study support the hypothesis that higher parental involvement is an indicator of better adolescent mental and physical health. These results are consistent with studies in Oman and larger studies across the Pacific Islands and Caribbean countries that explored trends in similar parameters of mental and physical health [42, 44, 51].

Childhood psychosocial development is influenced by parents. However, this influence decreases during adolescence while conflict peaks, leading to an erosion of parental power and an increased focus on parental psychological control that may impact child-adolescent development [7]. Results from this study are comparable to studies from other countries, such as India which showed higher levels of poor mental health in children with less parental involvement, and Lebanon, Palestine, Jordan, Syria, and Vietnam which showed that lack of parental understanding was associated with higher incidence of suicidal ideation/thinking [18, 19, 25, 30]. Those studies also used the GSHS databases for the corresponding countries reporting parental involvement using the same questions utilized in this study.

Moreover, similar to our results, parental involvement (specifically parental understanding and parental monitoring) was associated with a reduced odds of being bullied or physically harmed among Vietnamese adolescents. This finding reinforces the idea that parental involvement can have a protective effect against school bullying [30]. In another study using GSHS data from Oman, parental support was identified as a protective factor associated with reduced school truancy [39].

With regards to physical health, this study’s findings suggest that parental involvement is associated with both improved nutrition and improved physical activity. Across the Eastern Mediterranean region, USA and Australia, consumption of fruits and vegetables has been found to be low in part due to less familial activities [5, 38]. Larger studies from the USA and Eastern Mediterranean countries showed higher family functioning was associated with lower BMI, with less sedentary behaviour and better nutritional habits [3, 41]. This suggests that health risk behaviour should be a focal target among adolescents in Oman to improve efficacy of health promoting initiatives. Evidence suggests that when parents act as the primary contacts for learning and implementing social constructs, adolescents are influenced both with regards to weight and health decisions across various socio-cultural backgrounds in the study population [47].

Parental involvement is a dyadic relationship, being dependent on both attention from parental figures and the willingness of the adolescent to partake in information exchange [10]. Previous studies suggest that when parental figures have higher levels of involvement and more knowledge of adolescent activities, this leads to eventual lower levels of risk taking behaviour [10]. This supports the results showing increased parental involvement was associated with less risky behaviour, especially substance use. Our study found lower rates of tobacco use among adolescents with higher levels of parental involvement. This is consistent with literature from In Finland where higher parental involvement was associated with lower rates of daily smoking among adolescents, as well as a multinational GSHS study which found parental involvement to be a protective factor against smoking [24]. This may be due to parental monitoring and a healthy parent–child relationship, which in turn allows for a more controlled environment protective against the influences of peer pressure [22, 33, 37].

The findings of this study have to be considered in the context of the family structure in Oman. The family unit is usually traditional—with strong ties to both Arab culture and Islam. This results in both an integrated support system and a large emphasis on fulfilling roles within the family. In general, parenting styles across the MENA region, and worldwide, range from strict to flexible. Previous studies show that this family structure style and resultant trends exhibited by adolescents is consistent throughout the Arab world [25, 38]. This may be due to differences in various households and have an association with differences in socioeconomic and socio-political statuses of parents. It was found that parents that defined themselves as more conservative demonstrated more rigid and controlling behaviours towards their children as compared to more liberal parents [1]. However, in recent years, the traditional family unit has been evolving due to changes in communication, culture and overall attitudes towards gender roles and hierarchy [4]. One study found that these changes were often found to be positive, leading to a more openness between parents and children [40]. Not only this, but studies have shown that increased parental involvement improves academic success and emotional functioning and has an overall better transition to adulthood [46]. Other studies have shown that increased parental involvement further leads to higher levels of positive participation in school environments—leading to improved mental and physical well-being [1, 30, 42]. In light of the ever-evolving nature of the adolescent-parent relationship in Oman, this study provides a novel perspective on the benefits of improving and fostering this connection.

The study comes with several limitations. One limitation is that variables were self-reported which might lead to under-/over-reporting due to potential recall bias (some questions required recall of behaviors over the past 30 days) and adolescents are reporting on potentially socially undesirable behaviors. Our definition of parental involvement might not be all- inclusive and may not cover all aspects of parental involvement in adolescent development. Comparisons of our findings to other similar studies might not be accurate as different studies may define variables differently and our study does not measure the quality of parental interactions. The cross-sectional nature of the study does not allow for identification of causal or temporal relationships between selected variables. This data is also from 2015, and although this was the most recent data from the GSHS repository, further behavioural changes may occurred since. However, there are several strengths to this study, including the use of a large representative study sample of school going adolescents in Oman which, in principle, allows our results to be generalizable to the broad population of adolescents in schools.

In future studies, we recommend exploring the parental perspectives of adolescent health and to further explore the bi-directional nature of the parent-adolescent relationship. We also recommend future studies that longitudinally the effectiveness of culturally adapted interventions that aim to improve parental involvement in adolescent behaviours, as well as differences in when significant parental engagement occurred. Awareness campaigns and interventions aimed to help improve the well-being of adolescents should put some emphasis on parents and their relationship with their children.

## Conclusion

In conclusion, this study examined how parental involvement can affect adolescent mental and physical well-being in Oman. We found that parental involvement is a significant protective factor against risky behaviour and poor mental and physical well-being. The results of this study could encourage parents to take a more active role in their adolescent’s lives to promote increased number of healthy behaviours, as well as potentially guide healthcare professionals to include parents when providing healthcare management.

## Data Availability

The dataset supporting the conclusions of this article is available in the Global School Health Survey repository. [https://extranet.who.int/ncdsmicrodata/index.php/catalog/548].

## Abbreviation

GSHS: Global School Health Survey
